# DosePatch: physics-inspired cropping layout for patch-based Monte Carlo simulations to provide fast and accurate internal dosimetry

**DOI:** 10.1186/s40658-024-00646-y

**Published:** 2024-06-26

**Authors:** Francesca De Benetti, Julia Brosch-Lenz, Jorge Mario Guerra González, Carlos Uribe, Matthias Eiber, Nassir Navab, Thomas Wendler

**Affiliations:** 1 Chair for Computer Aided Medical Procedures and Augmented Reality, Technical University of Munich, Garching, Germany; 2 Department of Nuclear Medicine, Klinikum Rechts der Isar, Technical University of Munich, Munich, Germany; 3 Department of Integrative Oncology, BC Cancer Research Institute, Vancouver, Canada; 4 Department of Diagnostic and Interventional Radiology and Neuroradiology, University Hospital Augsburg, Augsburg, Germany; 5 Institute of Digital Medicine, University Hospital Augsburg, Neusaess, Germany; 6 Clinical Computational Medical Imaging Research, University of Augsburg, Augsburg, Germany

**Keywords:** Monte Carlo simulations, Dosimetry, SIRT, Dose kernels, Organ-S-values

## Abstract

**Background:**

Dosimetry-based personalized therapy was shown to have clinical benefits e.g. in liver selective internal radiation therapy (SIRT). Yet, there is no consensus about its introduction into clinical practice, mainly as Monte Carlo simulations (gold standard for dosimetry) involve massive computation time. We addressed the problem of computation time and tested a patch-based approach for Monte Carlo simulations for internal dosimetry to improve parallelization. We introduce a physics-inspired cropping layout for patch-based MC dosimetry, and compare it to cropping layouts of the literature as well as dosimetry using organ-S-values, and dose kernels, taking whole-body Monte Carlo simulations as ground truth. This was evaluated in five patients receiving Yttrium-90 liver SIRT.

**Results:**

The patch-based Monte Carlo approach yielded the closest results to the ground truth, making it a valid alternative to the conventional approach. Our physics-inspired cropping layout and mosaicking scheme yielded a voxel-wise error of < 2% compared to whole-body Monte Carlo in soft tissue, while requiring only $\approx$ 10% of the time.

**Conclusions:**

This work demonstrates the feasibility and accuracy of physics-inspired cropping layouts for patch-based Monte Carlo simulations.

## Background

One of the treatment options for hepatocellular carcinoma (HCC) is Yttrium-90 (^90^Y) selective internal radiation therapy (SIRT). Historically, the treatment activity of the ^90^Y-loaded microspheres was determined based on the body-surface area of the patient [1]. The use of personalized approaches in SIRT was recently demostrated to increase the overall survival [2]. A certain personalization is based on the ^99m^Technetium-MAA SPECT/CT acquired for the treatment simulation [3]. The dose to the tumors is desired to be as high as possible, while keeping the dose to healthy perfused liver below safety limits [2]. This approach computes only mean compartment (tumor and healthy liver, respectively) doses, while a voxel-wise method would offer the advantage to consider the heterogeneities of the dose distribution within the liver and the tumor. Despite the proven benefits, there is no wide consensus about the introduction of personalized dosimetry in clinical practice due to the many sources of uncertainties (e.g., the limited spatial resolution of Nuclear Medicine images and possible errors in registration) [4] and the additional acquisition of data usually required to run such calculations (i.e., additional 3D imaging) [5], among others.

Following the Medical Internal Radiation Dose Committee (MIRD) formalism [6], many of the currently available software for dosimetry (such as OLINDA/EXM,^1^ IDAC-Dose [7], MIRDcalc [8]) base their calculations on human phantoms and therefore provide quick and accurate mean organ doses but lack of voxel-level dosimetry. Moreover, they consider homogeneous source and medium distributions. Few commercial software (e.g., MIM^2^ or DosePlan^3^) offer voxel-level dosimetry by convolving a dose-point kernel (DPK) with a Nuclear Medicine volume [9], and therefore take into consideration heterogeneous activity distributions. DPK are pre-calculated for specific tissues (often water-equivalent), and different approaches have been proposed to correct for differences in actual voxel density and DPK density, such as a CT-based density weighting on the 3D dose image [10, 11]. Also, some groups have modelled tissue heterogenity by using DPK for different tissues [12, 13].

Monte Carlo (MC) simulation is still considered the gold-standard for personalized dosimetry [14] since it takes into account the heterogeneity of the tissue density and chemical composition, as well as heterogeneous activity distribution. MC dosimetry is based on simulations that accurately model the emission, transport and interactions of primary and secondary particles emitted by the radioactive source within the surrounding tissues [15]. This comes at the cost of high computational burden and long simulation times from several hours to days, making it inappropriate for the use in clinical routine.

Cropping the input volumes into smaller patches would allow to run multiple simulations at the same time. MC simulation on patches was employed by Lee et al. [16] as ground truth of their neural network for dose rate estimation from CT and PET. Patch-based methods require a final step, called mosaicking, in which the patches are stitched together to generate the final volume. When dealing with patch-based dosimetry, the cropping and the mosaicking of the patches need to be performed in a way that respects the physics of the radiation transport and its interactions with matter.

The main contributions of this paper are:

- The introduction of a physics-inspired cropping layout for patch-based MC-dosimetry (CL3) that provides a fast but accurate approach to generate 3D dose maps
- The comparison of the proposed cropping layout (CL3) with a naive one (CL1) and the one proposed by Lee et al. [16] (CL2).
- The evaluation of the performance in terms of computation time and accuracy of these cropping layout approaches with conventional dosimetry methods.

To the best of our knowledge, this is the first work comparing patient-individual MC absorbed dose simulation, dose-kernel-based dosimetry, patch-based MC simulation using different cropping layouts, and the conventional MIRD formalism using organ S-values for ^90^Y SIRT.

## Methods

### Dataset description

The dataset consists of 5 patients (59–86 years, 4 males, 1 female) with an unifocal HCC treated with ^90^Y SIRT at Klinikum rechts der Isar (Munich, Germany) between February 2016 and June 2019. Each patient has a 1-bed position ^90^Y Bremsstrahlung SPECT/CT that was acquired on the same day of the liver SIRT. The CT was low-dose (24–27 mA, 130 kVp) with 2.5 mm slice thickness and pixel spacing of 0.74–0.98 mm in *x* and *y* direction. The SPECT was acquired with two heads, resulting in 64 projections of 30 s each. It was reconstructed using OSEM (8 iterations, 8 subsets), corrected for attenuation using the CT, and filtered with a 10 mm Gaussian filter to an isotropic resolution of 9.59 mm. The SIRT treatment activity ranged between 0.79 and 1.51 GBq; the right lobe was treated in three patients, while the left was treated in two.

A self-calibration approach was used to convert the SPECT images from counts to units of Becquerel. The patient-specific calibration factor was determined by dividing the ^90^Y therapy activity, which was decay corrected to the image acquisition start time, by the total counts in the SPECT image [17].

### Preprocessing

The CTs were resampled to the bremsstrahlung SPECT matrix of 64 $\times$ 64 $\times$ 42 voxels with isotropic voxel size of 9.59 mm. The field of view of our input volumes was variable, but in general it included the abdomen and the pelvis. An experienced reader annotated the liver and the lungs in the CT of the bremsstrahlung scan. In the following, we will consider liver, lungs and the remainder of the body (ROB) as our volumes of interest (VOIs).

To be able to compute the absorbed dose, we generated a 3D time integrated activity (TIA) map per patient. The TIA gives the information about number of decays that take place in each voxel during the duration of the therapy. On the assumption that the microspheres are permanently trapped in the liver tissue, only the physical decay of ^90^Y needs to be considered to generate the TIA map. The 3D TIA map was obtained by applying the following to each voxel of the bremsstrahlung SPECT:

1 $$\begin{aligned} TIA = \int _0^\infty A(t) dt = \frac{A(0)}{\lambda } = \frac{A(T) e^{\lambda T}}{\lambda } \end{aligned}$$

with *A*(0) being the activity per voxel at time of the application of microspheres ($t=0$), *A*(*T*) being the activity per voxel at time of the bremsstrahlung SPECT acquisition ($t=T$) and $\lambda$ the decay constant of ^90^Y defined as $\lambda = \frac{ln2}{t_{1/2}}$ ($t^{^{90}Y}_{1/2} = 64.2$ h [18]).

### Monte Carlo benchmarking

All the MC simulations were run in the GATE platform (version 9.2, with GEANT4 11.0) [14, 19, 20], with the MersenneTwister random seed. The simulated physical processes were those included in the emstandard_opt3 physics list and the RadioactiveDecay. To benchmark our code, we computed the S-value for a sphere of water with mass equal to 60 g and we compared the results with IDAC’s reference values [7].

### The conventional Monte Carlo approach (DoseMC)

As a reference, we simulated the 3D absorbed dose per patient with GATE, using the TIA map and the CT scan as inputs. The density and materials tables proposed by Schneider et al. [21] were used to convert Hounsfield Units (HU) to materials and densities to be used by GATE. The source (defined by the TIA map) was simulated with the built-in ion source of GATE. The range thresholds for particle tracking were set to 0.01 mm in the patient region, which corresponded to an energy threshold of 15 keV in soft tissue for electrons.

We split the simulation into ten jobs with $5\times 10^{7}$ simulated particles each, for a total of $5\times 10^{8}$ simulated primaries. The output of each job was a 3D volume with the same matrix and voxel size of the volumes used as inputs.

Finally, the individual simulations were merged using the weighted average and dose uncertainty computed according to Chetty et al. [22]. Following the approach of Brosch-Lenz et al. [11], the output is multiplied by the sum of the TIA and divided by the number of particles to account for the difference between the total number of simulated particles ($5\times 10^{8}$) and the real ones (equivalent to the sum of the TIA map).

### The kernel approach (DoseKernel)

We estimated the absorbed dose using the DoseKernel approach by convolving the TIA map with voxel S-values (VSVs) representing the absorbed dose distribution per decay. The VSVs were generated with GATE by placing the source of the radionuclide in the central voxel of a matrix of ICRP soft tissue [23, 24] and simulating $10^8$ primaries. The ^90^Y VSV had a size of 25 $\times$ 25 $\times$ 25 voxels with 9.59 mm isotropic voxel size (same as the SPECT images). The particle range thresholds were set to 0.001 mm. In a separate step, the CT was used to build a patient-specific 3D density map using a HU-to-density conversion curve. Finally, by multiplying the output of the convolution by the density map, we took into account the difference in density between the ICRP soft tissue used in the simulation of the VSVs and those of the different regions of the human body [11].

### The IDAC approach (DoseIDAC)

To include the organ S-value approach into the comparison, we used IDAC Dose [7] to compute mean doses in the volumes of interest (VOIs). Mean TIA of liver, lung and ROB were used as inputs for IDAC. The VOI volumes were then converted to the actual masses of the patient’s organs to account for any differences between the patient’s organ mass and the mass of the ICRP reference phantom. The average organ densities per VOI were taken from the 3D density map, as generated in the DoseKernel approach.

### The patch-based Monte Carlo approach (DosePatch)

In the DosePatch approach, one TIA map patch and the corresponding CT patch were used as input of the GATE simulation. Similarly to DoseMC, the source (defined by the TIA map patch) was simulated with the built-in ion source of GATE, the cuts were set to 0.01 mm, and the GATE simulation returned a 3D dose patch with the same size and voxel size of the patches used as inputs. In the following, we will use 8 patches per volume, with $6.25\times 10^7$ simulated particles per patch, which accounts for the same number of simulated particles per patient as in DoseMC (namely, $5\times 10^{8}$), thus ensuring comparability between the two methods.

**Fig. 1:**
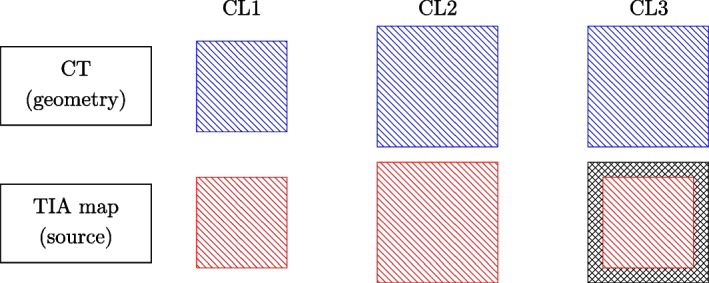
Different cropping layouts (CL) for the DosePatch approach

**Fig. 2:**
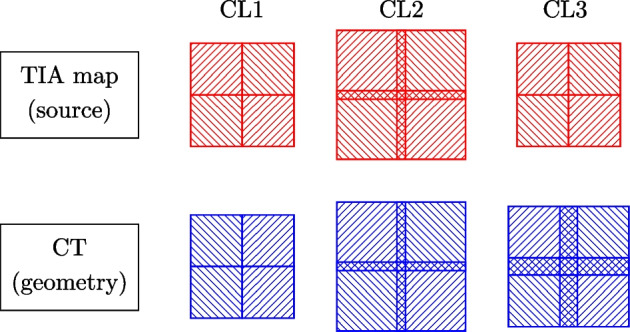
Mosaicking approach for all the three cropping layouts

The CT and the TIA map were first zero-padded to ensure that an integer number of patches could be fitted in the volumes. Then, the volumes were cropped into patches following three different cropping layouts (CL), as shown in Fig. 1. Finally, to return the whole-body 3D absorbed dose, the dose patches were mosaicked, taking care of modelling correctly the physics of radiation at the border of the patches (see Fig. 2). While performing the mosaicking, each of the patches is multiplied by the sum of the patch TIA and divided by the number of simulated primaries, following the same approach used for DoseMC [11].

The details of the three CLs, the mosaicking approaches, and the physics behind them are explained in the next paragraphs.

#### Cropping layout 1

The TIA map and the CT patches had the same size (32 $\times$ 32 $\times$ 32 voxels) and were cropped without overlap.

At the mosaicking step, the patches were simply placed one next to the other in the same order in which they were cropped. We implemented this set-up only as a naive approach. This layout is physically wrong since particles reaching the border of the TIA patch are completely lost instead of depositing energy on neighboring patches or being back-scattered there. Therefore they do not contribute to any absorbed dose, resulting in a lower dose at the border of the patches.

#### Cropping layout 2

This set-up was inspired by the work of Lee et al. [16]. The TIA map and the CT patches had the same size (40 $\times$ 40 $\times$ 40 voxels) and were cropped with overlap of 4 voxels (equivalent to 38.36 mm) on each side.

**Fig. 3:**
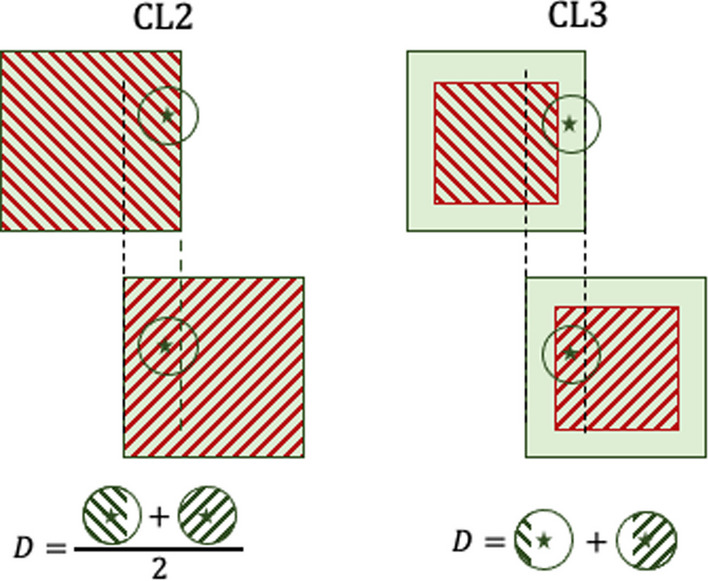
The green square represents the dose patch, the red stripes represent the source patch (in the case of CL2 they have the same size), the green circle represents the region where the radiation emitted by the source patch contributes to the dose in the green star. The green circles on the bottom represent the contribution to the absorbed dose in the green star coming from the two patches, with the corresponding math used in the mosaicking. Considering the contribution to the dose in the point marked by the green star: *CL2*—The green circle is fully inside the source/dose patch in the right patch, but it is partially outside the left patch. The total dose *D* is computed as the average of the contributions from left and right patch, but this results in an underestimation because part of the green circle in the left patch is outside of the source patch. *CL3*—The green star is outside the left source patch, but it is inside the right source patch: the contribution to the dose in the green star is roughly 30% from the left and 70% from the right dose patch. We computed *D* by summing the two contributions, which leads to the correct dose estimation

During mosaicking, we considered the fact that the TIA map, the CT and the dose patches were overlapping. Therefore, in the region where the TIA map patches overlap, the contribution to the dose came from $N=\{2, 4, 8\}$ source patches depending on whether the overlap occurs at the border of two patches or the intersection of four patches (see Fig. 2). To compensate for this, the whole-body 3D dose in the overlapping region was computed as the average of the *N* overlapping dose patches. This approach is better than CL1 since less particles are lost, but it is still physically incorrect. Every voxel in the dose map which is in the center of the overlapping region, has contribution from voxels belonging only to the overlapping region. The contributions to voxels close to the border of the overlapping region come from primaries originating inside (as before) but also outside of the overlapping region. As a result, the mosaicking approach for CL2 is only able to partially address the problem of CL1. A visual explanation can be found in Fig. 3.

#### Cropping layout 3

**Fig. 4:**
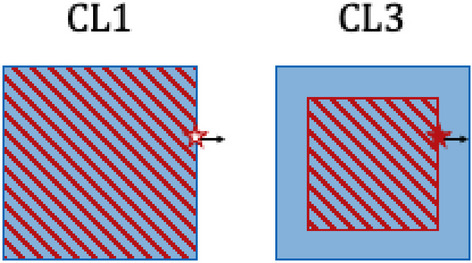
The blue square represents the geometry patch and the red stripes represent the source patch (in the case of CL1 they have the same size). We now consider radiation emitted by the point marked with the red star, where the black arrow represents the maximum penetration depth of the radiation. In CL1, the arrow ends outside the geometry patch, and therefore the contribution to the dose coming from the border of the source patch is lost. Also, backscattered radiation gets lost. On the other hand, the bigger size of the geometry patch in CL3 ensures that the arrow ends inside the geometry patch

The TIA map patch had size 32 $\times$ 32 $\times$ 32 voxels and was cropped without overlap, and the CT had size 40 $\times$ 40 $\times$ 40 voxels, with 4 voxels overlap. The TIA map patch was zero-padded to match the size of the CT patch. We proposed CL3 to overcome the drawbacks of CL1 and CL2. CL3 solves the problem of losing primaries (as in CL1) by using a CT patch that is bigger than the TIA map patch. This allows primaries being emitted on the border of the TIA map patch to still end up inside the CT patch (see Fig. 4). At the same time, the fact that the TIA map patches do not overlap (or they overlap only in the region in which they are zero-padded) ensures that every voxel in the dose map which in the overlapping region had contribution coming exactly from one (and only one) TAC map patch.

During the mosaicking process, we took into consideration that the CT and the dose patches were overlapping, whereas the TIA map patches were not. Therefore, in the region where the dose patches overlap, the contribution to the dose came from only one source patch. The whole-body 3D dose in the overlapping region was then computed as the sum of the $N=\{2, 4, 8\}$ overlapping dose patches. A visual explanation can be found in Fig. 3.

In CL2 and CL3, the number of overlapping voxels (*OV*) was selected to be higher than the range (*R*) of the $\beta ^{-}$ particles in soft tissue of the isotope in use ($R^{^{90}Y}_{\text {max}} = 11.3$ mm [25]). Specifically, we defined the minimum number of overlapping voxels as $OV_{min} =~\Big \lceil {\frac{2\cdot R}{v}}\Big \rceil$, where *v* is the voxel size. For implementation reasons, the number of overlapping voxels was chosen to be the closest even number to $OV_{min}$.

Finally, we chose the above patch sizes to ensure that all the CLs resulted in the same number of patches (8 per volume). This was required to perform a proper evaluation, having matching overlapping regions in the different CLs.

### Evaluation

The evaluation of the different methods was carried out by comparing their mean absorbed doses in different VOIs (lungs, liver and ROB). We reported percentage differences (PD) on mean VOI doses between DoseMC ($D_{MC}$) and the three other approaches (DosePatch, DoseKernels and DoseIDAC):

2 $$\begin{aligned} PD = \frac{|D_{MC} - D|}{D_{MC}}~\cdot 100 \end{aligned}$$

where *D* is mean VOI dose computed with one of the other approaches.

We visualized the PD in the VOI as boxplots^4^, as well as histograms to give a more detailed interpretation of the differences within the VOI.

For DosePatch we observed a VOI absorbed dose close to the one on DoseMC (see Section "Dosimetry approaches: VOI absorbed dose"). Therefore, we deepened our analysis by evaluating the performance of the three DosePatch approaches at a voxel level, in order to obtain a better understanding of how similar to DoseMC the resulting absorbed whole-body (WB) dose volumes are. Percentage difference maps were used to visualise the PD at voxel-level.

The focus of our analysis was the mean voxel-wise PD in the overlapping region, but, for completeness, we also reported it in the non-overlapping region and in the WB. Due to the different designs of the CLs, the dimension of the overlapping region was different (see Fig. 2). Therefore, we chose to evaluate the voxel-wise PD in the area in which all the three CL were overlapping (or there was the border in case of CL1), which is the overlapping region of CL2.

In all evaluations, the comparison was carried out only in soft tissue (i.e., the air around the patient, in the bronchi and inside the bowel was not considered).

For the voxel-level visualization, we used 2D PD maps, as well as 1D PD line plots. While the former give a global interpretation of the results, the latter shows in detail the difference between the CLs focusing on the overlapping region.

As an additional evaluation metric, we report the so-called “efficiency factor” (*EF*), which is defined as: $EF = \frac{1}{ T\,\cdot \, U^2}$, where *T* is computation time and *U* is the percentage statistical uncertainty [26].

## Results

### Monte Carlo benchmarking

Our benchmarking yielded a S-value for the 60 g sphere of water of $2.2689\cdot e^{-12}$ Gy/decay, whereas IDAC reported $2.2679\cdot e^{-12}$ Gy/decay [7]. Therefore, the benchmarking resulted in a percentage difference of only $-$ 0.04%.

### Dosimetry approaches: VOI absorbed dose

**Fig. 5:**
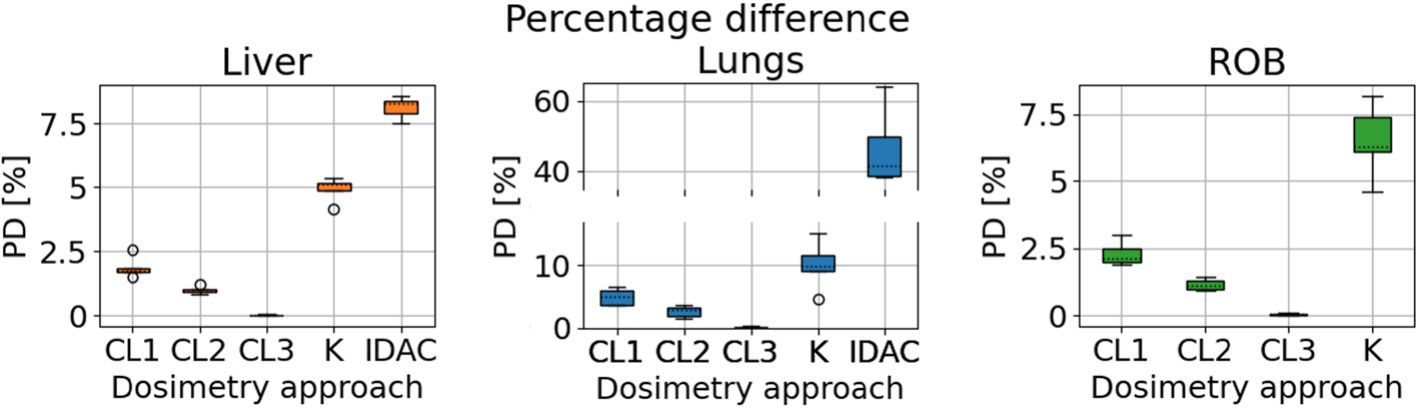
Percentage difference (PD) of the four dosimetry approaches on mean VOI doses, when compared with DoseMC. From left to right: DosePatch CL1, CL2, and CL3, DoseKernel (denoted by “K”), DoseIDAC (“IDAC”)

The VOI percentage difference of the three CLs of DosePatch, DoseKernel and DoseIDAC versus DoseMC are reported as boxplots in Fig. 5. The approach with the lowest PD compared to DoseMC was DosePatch-CL3 (0.01 ± 0.01% in the liver), whereas DoseIDAC returned the largest (46.52 ± 9.73% in the lungs). However, with the same approach, the average PD in the liver was only 8.12 ± 0.40%. A similar phenomenon (i.e. highest PD in the lungs and lowest in the liver) appeared in CL1, CL2 and CL3 and in DoseKernel.

### Dosimetry approaches: voxel-wise absorbed dose

As shown in Table 1, CL3 had the lowest percentage difference when compared to DoseMC in the overlapping region (1.53 ± 0.24%), whereas the highest was found in the overlapping region of CL1 (6.39 ± 0.51%). In the non-overlapping region, the PD was similar among the three CLs.

**Table 1:** Voxel-wise percentage difference (mean ± std) [%]: DosePatch versus DoseMC

Percentage difference	CL1 (%)	CL2 (%)	CL3 (%)
WB	3.72 ± 0.27	2.80 ± 0.28	1.90 ± 0.26
Overlapping	6.39 ± 0.51	3.66 ± 0.43	1.53 ± 0.24
Non-overlapping	2.18 ± 0.27	2.30 ± 0.28	2.12 ± 0.27

**Fig. 6:**
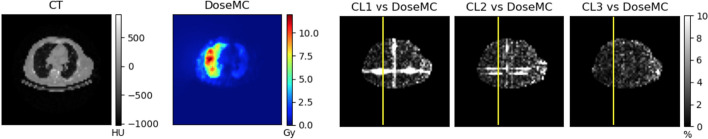
From left to right: CT, DoseMC, PD maps of DosePatch-CL1, CL2, and CL3 when compared to DoseMC

Figure 6 shows one selected slice of percentage difference maps of CL1, CL2 and CL3 (the CT and DoseMC slices are included for completeness).

**Fig. 7:**
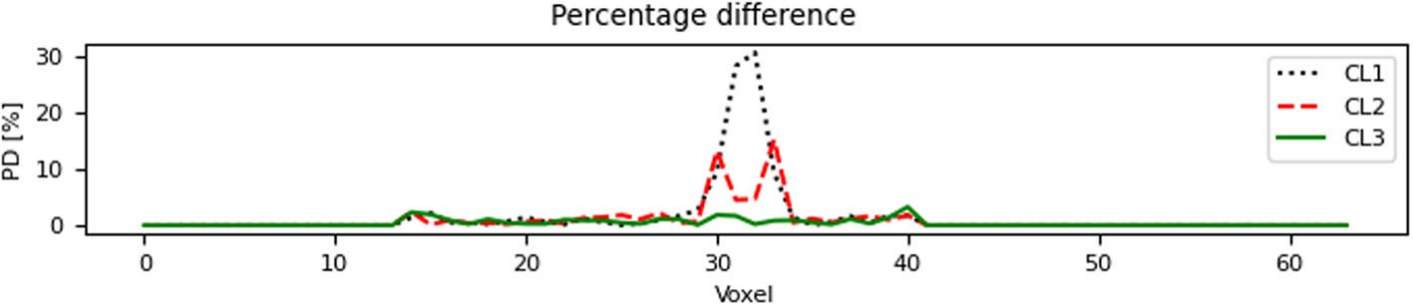
Percentage difference of the different cropping layouts of the DosePatch approach as line profile plots along the yellow line depicted in Fig. 6, when compared with DoseMC

The line profile plots in Fig. 7 focus on the percentage difference along the yellow line depicted in Fig. 6. It can be seen that CL1 had a spike of about 30%, whereas CL2 had two smaller spikes around 15%. The spikes corresponded to the border of the patch in CL1 and the overlapping region in CL2, respectively. Outside the overlapping region, CL1 and CL2 had a PD around 5% or lower. In the case of CL3, the percentage difference was uniformly lower than 5% along the whole line, including in the overlapping region.

The difference in the WB absorbed dose between DoseMC and DosePatch-CL1 and DosePatch-CL2 is statistically significant (*p* $<\,10^{-4}$), whereas with DosePatch-CL3 it is not (*p* = 0.099).

### Uncertainty

**Fig. 8:**
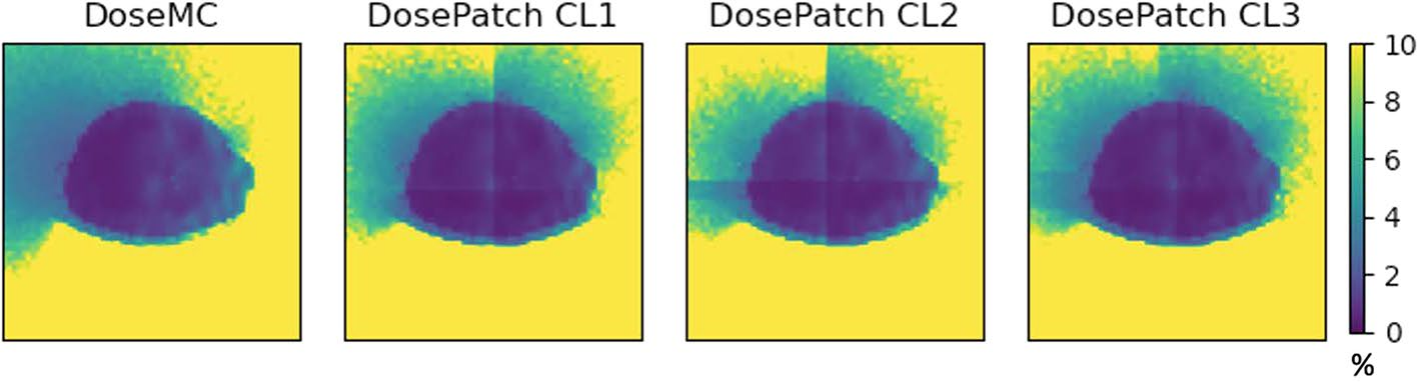
Percentage statistical uncertainty for the DoseMC approach and the DosePatch approach with the three cropping layouts

The average statistical uncertainty in the whole body was $\in$ [1.32, 1.68]%, which is consistent with previously published results [27], with the maximum found in DoseMC and the minimum in DosePatch-CL3. If we consider only the the lungs and liver, the average statistical uncertainty was $\in$ [0.71, 0.82]%, with the maximum found in DosePatch-CL2 and the minimum in DoseMC (Fig. 8).

### Computation time

Computing a full dosimetry report with IDAC took a few minutes, while with DoseKernel it required only a few seconds, after the kernel became available (its simulation with GATE can take up to 76 h on a single CPU, but it is computed only once). One DoseMC simulation with $5\times 10^7$ particles can take up to 35 h, for a total computation time of $\approx$ 350 h (10 simulations—$5\times 10^8$ particles in total). In the case of DosePatch, one patch took $\approx$ 250 min (with $6.25\times 10^7$ simulated particles), yielding a total computation time of $\approx$ 34 h (8 patches—$5\times 10^8$ particles in total).

The efficiency factor of DoseMC is 1.01$\times 10^{-3}$, whereas all the DosePatch approaches have an higher *EF*, with the maximum found in DosePatch-CL3 ($EF^{\text {CL3}} = 1.72\times 10^{-2}$), confirming that our proposed approach is superior to DoseMC.

### DosePatch: scalability

The DosePatch approach can be implemented with any number of patches, as long as the requirements presented in Section "The patch-based Monte Carlo approach (DosePatch)" are satisfied. We applied the DosePatch-CL3 approach with 18 patches (of size 32 $\times$ 32 $\times$ 32 for the CT and 24 $\times$ 24 $\times$ 24 for the TIA map), using $2.78 \times 10^7$ particles per patch to ensure a total number of particles equal to DoseMC.

In the VOI, the PD versus DoseMC is comparable to the one of DosePatch-CL3 with 8 patches (DosePatch-CL3^8^). The voxel-wise PD in the WB was slightly higher (2.61 ± 0.28% for DosePatch-CL3^18^ and 1.90 ± 0.26% DosePatch-CL3^8^). In terms of uncertainty of the MC simulation, it was 2.48 ± 0.10% for DosePatch-CL3^18^, which is higher than the one reported for DosePatch-CL3^8^ and DoseMC, but still comparable with previously reported results [27]. Finally, in terms of computation time, on average the MC simulation of one DosePatch-CL3^18^ took $\approx$ 140 min, for a total of $\approx$ 41 h for the simulation of one WB volume, which is slightly higher than the computation time for DosePatch-CL3^8^.

## Discussion

### Dosimetry approaches: VOI absorbed dose

The DosePatch approach was the one in closest agreement with the reference DoseMC, with a mean VOI absorbed dose percentage difference lower than 2.85% in WB, with it decreasing to $0.06\%$ in CL3.

In the liver and the ROB, the three DosePatch approaches resulted in a percentage difference $\in [0.02, 2.29]\%$, whereas for DoseKernel PD was 4.92% and 6.52%, respectively, and for DoseIDAC it was 8.12% in the liver. Previously reported values are consistent with our findings. For example, Grimes and Celler [28] reported a maximum percentage difference in soft tissue between DoseMC and DoseIDAC of $-$ 6.2% (for ^177^Lu) and of 7.4% (for ^99m^TC) when DoseKernel (without density correction) was used. Moreover, Kim et al. [29] found an average PD of 5% in the kidneys when comparing DoseMC and DoseIDAC and of 1.23% if using DoseKernel (both with ^177^Lu).

For each dosimetry approach, the lungs resulted in the highest PD among the VOIs (up to 46.51 ± 10.88% in DoseIDAC, 10.10 ± 3.90% in DoseKernel and 4.98 ± 1.32% in DosePatch-CL1), consistently with what was reported by Lee et al. [12] while testing their multiple DPKs approach. The large PD observed for DoseIDAC can be attributed to the challenge of accurately modelling the heterogeneous materials in the lungs, which include vessels, bronchi and air. The mass scaling of the S-values was not able to fully account for these differences. In addition to this, DoseIDAC introduced even larger errors due the phantom-derived organ S-values [27, 28] and the assumption of homogeneous activity distributions in the organs. For the DoseKernel approach, the voxel-wise density weighting aided in reducing the PD, but still the particle transport across very heterogeneous materials could only be accurately accounted for in the MC-based simulations (DoseMC and DosePatch). Compared to the Collapsed Cone superposition method proposed by Sanchez et al. [13], our DosePatch-CL3 returns a smaller percentage error in the lungs (0.15 ± 0.11, whereas their reported PD is $-$0.8 in [13]).

### Dosimetry approaches: voxel-wise absorbed dose

**Fig. 9:**
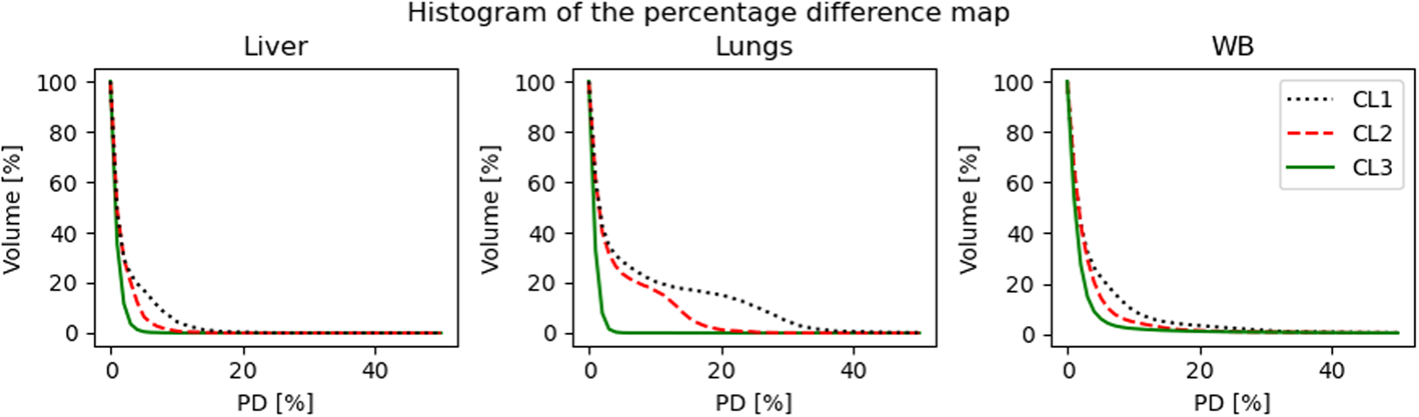
Histogram of the percentage difference map for one selected patient. In the histograms of liver and WB, the three lines are close, with solid green line (CL3) being the lowest one. On the other hand, for the lungs, the difference between the solid green line and the other two is larger. Indeed, for CL3 the majority of the voxels in the lungs have a PD lower than 10%

Consistently with the results for the VOI, CL3 had the lowest voxel-wise PD among the DosePatch approaches in WB and in overlapping and non-overlapping regions when evaluated at voxel-level. The PDs of the three different investigated cropping layouts were generally small compared against the gold-standard of the MC simulation. Figure 9 displays histograms visualizing the frequencies of PD map values for the three CLs.

Grimes and Celler analysed the voxel-wise percentage difference between DoseMC and DoseKernel (without density correction) and found it to be lower than 6.1% in soft tissue [28]. The DoseKernel method applied by Brosch-Lenz et al. [11] for bone tumors (with ^177^Lu) returned an average percentage difference versus DoseMC at voxel level of $-$ 5 ± 1%. Lee et al. [12] reported a voxel-wise PD lower than 5% in their multiple DPK approach, but with significantly higher (up to 40%) error on lungs boundaries, which we did not observe.

DosePatch-CL3 returned the best results because it was designed to ensure that no relevant radiation is lost during the simulation, as it happens in CL1, and that all dose contributions are correct in contrast to CL2. CL2 was introduced by Lee et al. [16] in their dose estimation approach employing a deep neural network (DNN). Despite it being theoretically more precise than CL1, it was not optimal, and, as indicated by our results, CL3 is superior to CL2. These effects are visible in Fig. 6, where in CL1 bright connected region corresponds to the wrong estimation of the dose. For CL2, the two white stripes with high PD in the overlapping region depict the fact that the computation in wrong only in the regions close to the border of the patch, and not in the whole overlapping regions. Finally, in CL3 the percentage difference map versus DoseMC is homogeneous, without visible bright regions.

These considerations apply only under the assumption that the overlapping region of CL2 and CL3 is bigger than the maximum penetration range of the $\alpha$ or $\beta$ emissions of the isotope in use, or in case of X-ray or $\gamma$-emitters, the distance in which at least 95% of the energy is deposited.

### Uncertainty

We also analysed how the PD at voxel-level compares to the uncertainty. In the non-overlapping region, the PD of DosePatch-CL1, DosePatch-CL2 and DosePatch-CL3 was comparable with the uncertainty of the GATE simulation of the patches, showing that a better result could be achieved only by increasing the number of simulated particles. The mean voxel-wise PD of DosePatch-CL1 in the overlapping region (6.39 ± 0.51%) was 5.5 times bigger than the uncertainty of the corresponding region (1.17 ± 0.15%). Similarly, in the case of DosePatch-CL2, the PD was 3.66 ± 0.43%, whereas the uncertainty was 0.95 ± 0.17%. This means that the PD of DosePatch-CL1 and DosePatch-CL2 in the overlapping region could not be due to the GATE uncertainty only, but it is also related to the design of the CLs. On the other hand, for DosePatch-CL3, the PD and the uncertainty were comparable (1.53 ± 0.24% and 1.09 ± 0.15%, respectively), showing a behaviour similar to what has been explained above for the non-overlapping region.

### Parallelization and computation time

The total time needed to achieve the final result of DoseMC, as well as all the simulations of DosePatch, depended on the number of simulations that can be run in parallel. Conventionally, MC simulations are parallelized by splitting the number of simulated particles in different runs. In DoseMC, we used this approach to simulate $5\times 10^8$ particles, split in 10 simulations with $5\times 10^7$ particles each.

In contrast, with DosePatch we proposed a different parallelization method. In the DosePatch approach, the parallelization is done by splitting the original field-of-view in eight different patches. Accordingly, the total number of primaries ($5\times 10^8$) was split homogeneously in each patch, thus accounting for $6.25\times 10^7$ primaries per patch.

Assuming to be able to run only one simulation at the time, the total time for DoseMC (10 simulations, with $5\times 10^7$ simulated particles each) was $\approx$ 350 h, and for DosePatch (8 patches, 8 simulations with $6.25\times 10^7$ simulated particles each) was $\approx$ 34 h. With this set-up, the computation time required by DosePatch was less than 10% of the one for DoseMC.

Villoing et al. [30] reported a computation time of $\approx$ 30 h for a ^90^Y GATE simulation of $10^8$ particles in the ICRP/ICRU female reference computational model. Sanchez et al. [13] reported an average computation time of 33 h in two ^90^Y clinical cases with $10^8$ particles simulated particles. We reported a computation time of $\approx$ 35 h for the simulation of $5\times 10^7$ particles. Despite the difference, which may be due to the differences in hardware set-up, image resolution and field of view, and GATE settings, the computation times are comparable.

### DosePatch: scalability

The results of Section "DosePatch: scalability" confirm that the choice of the number of patches in the DosePatch approach is not critical. However, further studies could be conducted to find the optimal number of patches in terms of PD, uncertainty, and computation time.

### Limitations

A limitation of this study is the small number of patients. This is due to the fact that WB MC simulations are time consuming and we therefore decided to include only 5 representative patient cases. However, we believe that our results will be transferable to other patient cases and will lead to the same conclusions as drawn in this work. Secondly, we kept the original voxel size of bremsstrahlung SPECT (isotropic 9.59 mm), resulting in low-resolution images used as input for all dosimetry analyses. However, the resolution and accuracy of bremsstrahlung imaging itself is limited and blurred by the range of the electrons before bremsstrahlung production. We therefore consider our approach being valid and applicable in the clinical setting of ^90^Y liver SIRT. Finally, we presented results only for ^90^Y, which is an effective example given the large penetration depth of the $\beta ^{-}$ in soft tissue. However, future analyses should expand upon this work and include other radioisotopes as well as other RPTs such as for example Lu-177-PSMA.

Even though outside of the scope of this work, an accurate comparison between the proposed DosePatch approach and the Collapsed Cone Superposition method [13] should be performed to highlight strengths and weaknesses of the two approaches on the same data since that method claims to be faster than a whole-body MC.

## Conclusions

In this work we evaluated three 3D dosimetry methods: two established approaches (DoseMC and DoseKernel), and a newly introduced patch-based Monte Carlo approach. We also included an organ-level dosimetry approach (DoseIDAC) as a reference due to its wide use in the clinical community. In particular, for DosePatch, we tested three cropping layouts with different combinations of patch overlap in the TIA maps and CT volumes.

We proposed CL3, a physics-inspired cropping layout, which is designed to account for the effect of the radiation at the border of the patches, by avoiding to lose any contribution to the dose (as in CL1) or to include more contribution than necessary (as in CL2). CL3 resulted in the lowest percentage difference among the methods we considered in this work, when compared to the conventional MC simulation (at both VOI- and voxel-level) while being $\approx$ 10 times faster. By reducing the computation time of such a large amount, the DosePatch approach shows the path to an efficient generation of 3D absorbed dose maps, allowing for a smoother integration of internal dosimetry in clinical practice. Moreover, this is an essential step towards the use of DNNs in dosimetry (like the one proposed by Lee et al. [16]), for which large databases of whole body patient-specific absorbed dose maps are needed. As such, we believe that this work provides useful information for the community of radioisotope- and brachytherapy as DNNs will play an increasingly important role in medical image analysis and, in particular, in dosimetry.

## Abbreviations

HCC: Hepatocellular carcinoma
SIRT: Selective internal radiation therapy
MIRD: Medical Internal Radiation Dose Committee
DPK: Dose point kernel
MC: Monte Carlo
CL: Cropping layout
VOI: Volume of interest
TIA: Time integrated activity
VSV: Voxel S-values
OV: Overlapping voxels
WB: Whole-body
PD: Percentage difference

## Footnotes

1 Hermes Medical Solutions, Sweden (https://www.hermesmedical.com).

2 MIM Software Inc, USA (https://www.mimsoftware.com).

3 SurgicEye GmbH, Germany (https://www.dose-plan.com).

4 The box extends from the first quartile to the third quartile of the data, with the horizontal line depicting the median. The whiskers extend from the box to 1.5 the interquartile range (difference between third and first quartiles). Data outside the range of the whiskers are considered outliers and shown as individual points.
